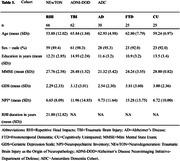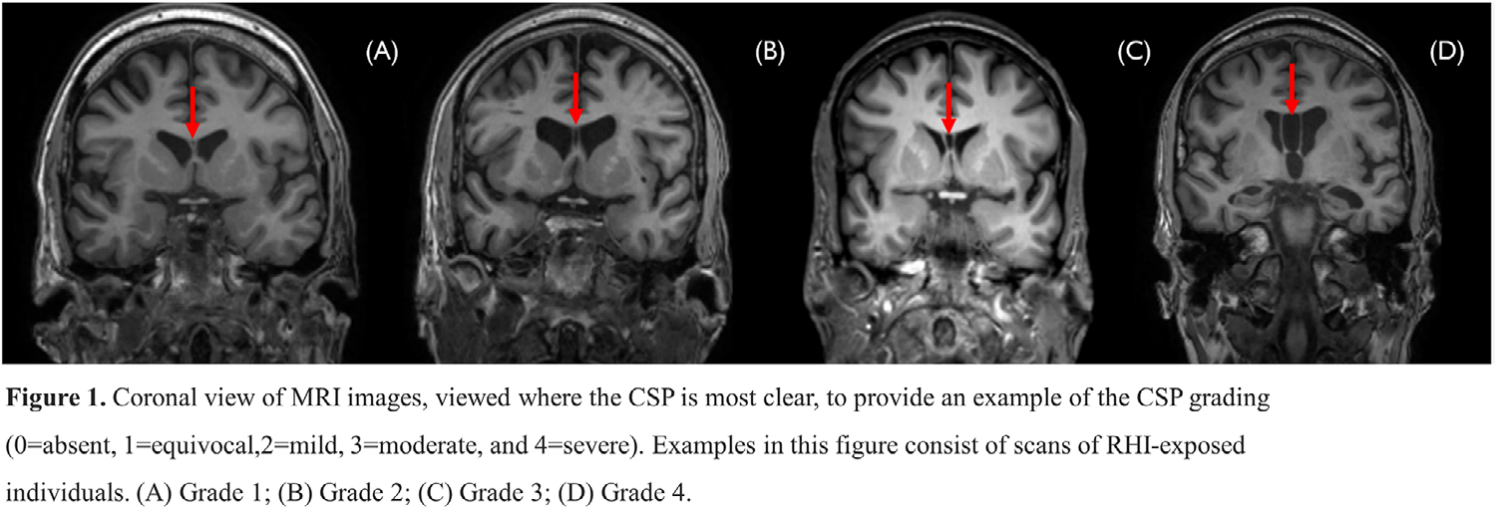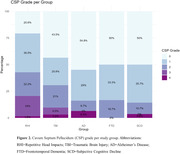# Cavum Septum Pellucidum (CSP) as a Neuroimaging Signature of Head Impact Exposure compared to Non‐exposed Patients with Neurodegenerative Disease and Controls

**DOI:** 10.1002/alz.093953

**Published:** 2025-01-09

**Authors:** Suzie Kamps, Hugo L. Hempel, Suzan van Amerongen, Hannah de Bruin, Fleur H.C. van der Linden, Frederik Barkhof, Philip Scheltens, Rik Ossenkoppele, Everard G.B. Vijverberg

**Affiliations:** ^1^ Amsterdam Neuroscience, Neurodegeneration, Amsterdam Netherlands; ^2^ Department of Radiology and Nuclear Medicine, Vrije Universiteit Amsterdam, Amsterdam UMC location VUmc, Amsterdam Netherlands; ^3^ Alzheimer Center Amsterdam, Neurology, Vrije Universiteit Amsterdam, Amsterdam UMC location VUmc, Amsterdam Netherlands; ^4^ Institute for Stroke and Dementia Research, Klinikum der Ludwig‐Maximilians Universität München, Munich Germany; ^5^ University College London, London United Kingdom

## Abstract

**Background:**

Cavum Septum Pellucidum [CSP] is commonly observed on neuroimaging in individuals exposed to repetitive head impacts [RHI] and in post‐mortem examination in Chronic Traumatic Encephalopathy [CTE]. A CSP is proposed as a potential biomarker for CTE, yet prevalence across neurodegenerative diseases and its clinical implications are largely unknown. We assessed CSP prevalence and clinical associations in RHI‐exposed individuals in comparison to veterans with a history of traumatic brain injury [TBI], individuals with a neurodegenerative disease (i.e. Alzheimer’s Disease [AD] or Frontotemporal dementia [FTD]) and Cognitively Unimpaired individuals [CU].

**Method:**

The group‐of‐interest, i.e., individuals exposed to RHI in contact sports or military service (n = 66), was compared against age‐ and sex‐matched ADNI‐DOD participants with TBI (n = 62) and non‐exposed participants of the Amsterdam Dementia Cohort (AD, n = 30; FTD, n = 25; CU, n = 25). Structural 3D brain MRI scans were visually rated on CSP grade (ranging 0‐4, Figure 1) according to established criteria by two independent raters without access to clinical information. A CSP scored at least grade 2 was considered abnormal. If scores between raters differed, scans were discussed to reach consensus. Inter‐rater reliability was assessed with Cohens’ weighted Kappa (κ). We investigated group differences in CSP grade as well as associations between CSP grade and neuropsychiatric symptoms (using the Neuropsychiatric Inventory [NPI]) and CTE probability (using the Traumatic Encephalopathy Syndrome [TES] criteria).

**Result:**

Inter‐rater reliability was substantial (κ = 0.712). Prevalence of an abnormal CSP differed between groups (χ2 = 11.72, p = .020). An abnormal CSP was observed most often in the RHI group (43%), followed by TBI (31%, OR = 0.589, p = .158), and significantly less in AD (16%, OR = 0.255, p = .014), FTD (17%, OR = 0.267, p = .029), and SCD (14%, OR = 0.222, p = .012) compared to RHI (Figure 2). Across groups, CSP grade was not associated with severity of neuropsychiatric symptoms (F = 1.7, p = 0.151). An abnormal CSP was observed more in RHI‐exposed individuals with probable (57%) or possible (55%) CTE compared to suggestive of CTE (36%) or no TES (36%).

**Conclusion:**

A CSP was more prevalent in RHI‐exposed individuals and veterans with TBI compared to patients with a neurodegenerative disease or CU individuals. Presence of a CSP on MRI may be indicative of head impact exposure, especially repetitive impacts.